# Sac1 links phosphoinositide turnover to cryptococcal virulence

**DOI:** 10.1128/mbio.01496-24

**Published:** 2024-07-02

**Authors:** Elizabeth A. Gaylord, Hau Lam Choy, Guohua Chen, Sydney L. Briner, Tamara L. Doering

**Affiliations:** 1 Department of Molecular Microbiology, Washington University School of Medicine, St. Louis, Missouri, USA; Duke University Hospital, Durham, North Carolina, USA

**Keywords:** *Cryptococcus neoformans*, mycology, phosphoinositide, PI4P, phosphatase, Sac1, virulence

## Abstract

*Cryptococcus neoformans* is an environmentally acquired fungal pathogen that causes over 140,000 deaths per year. Cryptococcal infection occurs when infectious particles are deposited into the lung, where they encounter host phagocytic cells. *C. neoformans* may be engulfed by these phagocytes, an important step of infection that leads to outcomes ranging from termination of infection to cryptococcal dissemination. To study this critical process, we screened approximately 4,700 cryptococcal gene deletion mutants for altered uptake, using primary mouse and human phagocytic cells. Among the hits of these two screens, we identified 93 mutants with perturbed uptake in both systems, as well as others with differences in uptake by only one cell type. We further screened the hits for changes in thickness of the capsule, a protective polysaccharide layer around the cell which is an important cryptococcal virulence factor. The combination of our three screens yielded 45 mutants, including one lacking the phosphatidylinositol-4-phosphate phosphatase Sac1. In this work, we implicate Sac1 in both host cell uptake and capsule production. We found that *sac1* mutants exhibit lipid trafficking defects, reductions in secretory system function, and changes in capsule size and composition. Many of these changes occur specifically in tissue culture media, highlighting the role of Sac1 phosphatase activity in responding to the stress of host-like conditions. Overall, these findings show how genome-scale screening can identify cellular factors that contribute to our understanding of cryptococcal biology and demonstrate the role of Sac1 in determining fungal virulence.

**IMPORTANCE:**

*Cryptococcus neoformans* is a fungal pathogen with significant impact on global health. Cryptococcal cells inhaled from the environment are deposited into the lungs, where they first contact the human immune system. The interaction between *C. neoformans* and host cells is critical because this step of infection can determine whether the fungal cells die or proliferate within the human host. Despite the importance of this stage of infection, we have limited knowledge of cryptococcal factors that influence its outcome. In this study, we identify cryptococcal genes that affect uptake by both human and mouse cells. We also identify mutants with altered capsule, a protective coating that surrounds the cells to shield them from the host immune system. Finally, we characterize the role of one gene, *SAC1*, in these processes. Overall, this study contributes to our understanding of how *C. neoformans* interacts with and protects itself from host cells.

## INTRODUCTION

*Cryptococcus neoformans* is a fungal pathogen that causes significant mortality in immunocompromised individuals, particularly those with HIV/AIDS (1). Despite recent advances in healthcare access and treatments, cryptococcal meningitis still leads to 19% of AIDS-related deaths (2). The interaction between *C. neoformans* and host phagocytes begins when environmentally acquired fungal cells are deposited in the lung. Subsequent phagocytosis may lead to fungal cell death and termination of the infection. However, in some cases, cryptococcal cells can withstand the pressures exerted by phagocytes to survive and proliferate within phagolysosomes (3–6). In this scenario, engulfment of the yeast likely enhances cryptococcal dissemination from the lung to other sites (7). Due to the pivotal role of this initial phase of cryptococcal pathogenesis, extensive efforts have been devoted to studying the interactions of *C. neoformans* with host phagocytic cells.

*C. neoformans* produces multiple factors which modulate its interactions with host cells and thereby provide protection from phagocytosis. These include the cell wall and surrounding polysaccharide capsule, complex glycan-based structures which change in size and composition in response to environmental conditions. Strains with perturbations of these structures often exhibit increased uptake by phagocytic cells (8, 9), demonstrating the importance of capsule for protecting against phagocytosis.

Various models have been used to interrogate cryptococcal-host interactions. These include invertebrate and vertebrate animals, amoeba, and primary and immortalized cells (3, 10–16). Genetic screens based on libraries of mutant host or fungal cells have identified gene products that modulate interactions of cryptococci with specific cell lines. In one example, Chun and colleagues subjected cryptococcal cells to insertional mutagenesis and identified mutations that affected their uptake by RAW264.7 macrophages (17). Srikanta et al. used RNA interference and high-throughput imaging to find host regulatory genes which govern the interactions of *C. neoformans* with THP-1 cells and discovered 25 fungal-specific regulators of phagocytosis (18). By applying high-throughput imaging to a *C. neoformans* gene deletion collection (19), Santiago-Tirado et al. identified 56 fungal genes that affect uptake by THP-1 cells (9). A common strength of these studies is the genetic tractability and accessibility of the immortalized cell lines used. However, such lines lack some features of primary host cells (20–22), which may limit these approaches. Screens may also be constrained by the availability and size of *Cryptococcus* mutant collections. For example, the last study cited screened a library of approximately 1,200 single gene deletion mutants (9), whereas much larger libraries are currently available, increasing our power to identify novel phenotypes.

This report incorporates several important departures from previous screens designed to elucidate *C. neoformans*-host interactions. First, we screened a collection of 4,692 single gene deletion mutants, representing over 80% of the non-essential genes in the cryptococcal genome. Second, we applied a flexible medium-throughput imaging method that avoids pitfalls of earlier studies, such as difficulty distinguishing adherent versus internalized yeast cells (23). Third, we screened using primary cells from both mouse (murine bone marrow-derived macrophages; BMDM) and human (monocyte-derived macrophages; HMDM) to take advantage of a model system while also examining the system of greatest clinical interest. Finally, we reasoned that many of our hits would have altered capsules, so we further tested mutants that demonstrated altered phagocytosis for changes in capsule thickness. Overall, we identified 45 deletion strains which exhibited altered uptake in both systems as well as altered capsule size.

While it is known that capsule material traffics at least in part through the secretory pathway (24), few proteins involved in this process have been characterized. To address this gap, we focused on mutants that might have aberrant capsule trafficking. One critical process in the regulation of secretion is the subcellular distribution of phosphoinositides (PI). Various cellular PI species are defined by the patterns of phosphorylation on their inositol head groups. These species are maintained at distinct cellular compartments by kinases and phosphatases, which specifically and reversibly phosphorylate and dephosphorylate inositol (25). In this way, PIs help define membrane identity and regulate membrane-protein interactions (25), thus playing important roles in secretory trafficking (26, 27). We therefore were particularly interested to note that one strain, which emerged as a hit in all three of our screens, lacks the gene *SAC1* (*CKF44_06080*). Sac1 is a phosphatase that is highly conserved in eukaryotes and acts in phosphoinositide metabolism (28). Our observation that cells lacking Sac1 are hypocapsular thus suggested that modulation of phosphoinositide turnover in the secretory pathway influences capsule synthesis. Below, we use biochemical, imaging, and molecular engineering approaches to establish the mechanism of this interaction, linking alterations in lipid homeostasis to the production of a critical cryptococcal virulence factor.

## RESULTS

### Fungal genes that influence interactions with host cells

To identify fungal gene products that affect interactions with host cells, we screened 4,692 *C*. *neoformans* single gene deletion mutants (19). In our assay, fungal cells were stained with Lucifer Yellow, opsonized with serum, and then incubated with primary phagocytes to allow engulfment. The samples were washed to remove unassociated cells and then stained with membrane-impermeant Calcofluor White to label externally associated fungi and propidium iodide to label phagocytic cell nuclei. We assessed fungal engulfment as the phagocytic index, or number of internalized fungi (stained with Lucifer Yellow but not Calcofluor White) per host cell (identified by nuclear staining) (23). We define the uptake score as the ratio of a mutant’s phagocytic index to that of wild-type (WT) *C. neoformans* (KN99).

**Fig 1:**
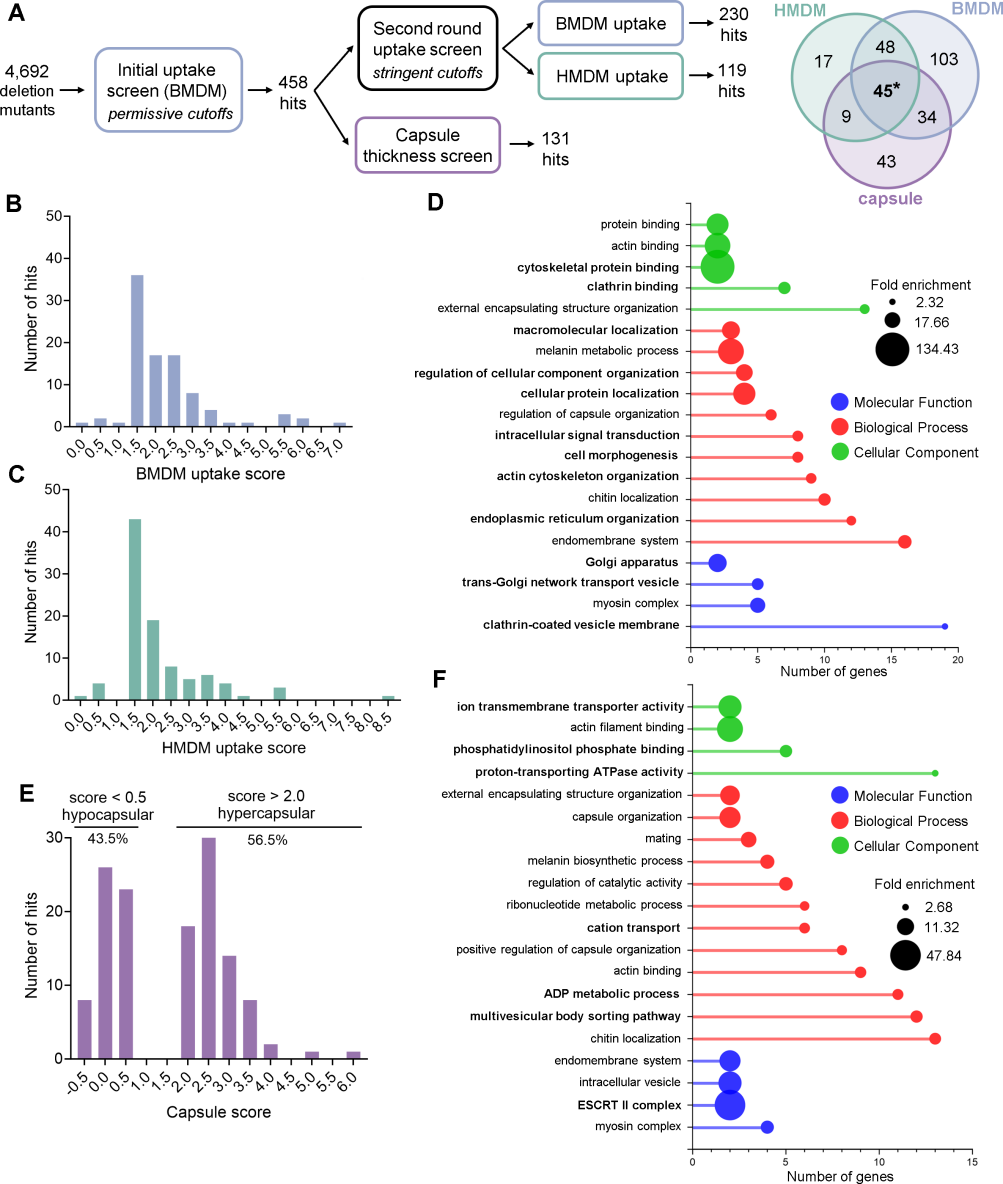
Screen results. (**A**) Schematic of screening workflow. Asterisk indicates *SAC1* in the diagram at right. (**B and C**) Uptake scores (as defined in the text) for the 93 mutants identified as hits when screened against both (**B**) BMDM and (**C**) HMDM. The value under each bar represents the center of the bin and 1.0 is the WT value. (**D**) Selected Gene Ontology (GO) terms that are enriched in the 93 dual hits from the uptake screen. X-axis, number of hit gene IDs which fall within each category. (**E**) Capsule score (capsule thickness relative to that of WT under inducing conditions) for 131 mutants identified as hits in the capsule screen, plotted as in (**B**) and (**C**). (**F**) Selected GO terms that are enriched in hits from the capsule screen, plotted as in (**D**). For panels D and F, bold text denotes categories uniquely enriched for each screen; note that not all categories are listed.

We first applied our assay to screen the interactions of *C. neoformans* with murine BMDM, normalizing to fungal cell number to account for differences in mutant growth rates (Fig. 1A). Because these deletion library plates do not include WT control cells, we initially compared strain uptake scores to the median for each plate and used permissive cutoffs to call roughly 10% of the strains as hits (Table S1A). We then subjected the 458 strains that we identified with increased or decreased uptake (Table S1B) to a second round of BMDM screening (Table S1C), this time comparing them to in-plate wild-type controls and using more stringent cutoffs to select hits (Fig. 1B; Table S1D). In parallel, we screened them using HMDM in place of BMDM (Fig. 1C; Table S1E and F).

Our second round of screening narrowed the initial hits to 93 mutants that significantly influenced host interactions in both BMDM and HMDM cells (Fig. 1A, overlap of red and blue circles; Table S1G). When we applied Gene Ontology (GO) analysis to the corresponding genes, we found that genes encoding cellular factors related to cytoskeletal interactions and intracellular protein localization and transport, among other categories, were particularly enriched relative to their abundance in the genome (Fig. 1D; see Discussion) (29, 30).

### Mutants with altered host interactions and capsule size

We were particularly interested in gene products that might act in capsule production because of the many outstanding questions that remain about this process (31). As the outermost layer of the cell, the capsule is fundamental in mediating cryptococcal interactions (7). Therefore, we hypothesized that our uptake screen hits would be enriched for mutants with altered capsule. To test this idea, we adapted an automated, fluorescence-based capsule quantification assay (32) to accommodate medium-throughput screening. Of the 458 first round hits from the uptake screen, 131 indeed showed capsule changes (Fig. 1A), with 57 strains identified as hypocapsular and 74 as hypercapsular (Fig. 1E; Table S2A and B). Validating the screen, this group contained multiple previously identified capsule mutants, including 16 that were hypocapsular and 3 that were hypercapsular. GO terms for the hits were particularly enriched for roles in capsule organization, as we expected, and for processes related to vesicular traffic, some of which were also enriched in our uptake screen hits. Uniquely enriched for the capsule screen hits were ion transport and phosphoinositide binding (Fig. 1F).

We identified 45 mutants which were hits in all three of our screens (Table S2C). The combination of perturbed interactions with phagocytes and altered capsule thickness of these strains suggested that the deleted genes would be important for virulence. We further speculated that the uptake phenotypes were related to surface changes in the cells, which might be mechanistically elucidated by analysis of the deleted genes. Of the 45 triple hits, 18 had been previously characterized. When we used homology to predict the subcellular localization of the remaining gene products, the largest group (8 of 27) was predicted to reside in the secretory pathway. We suspected these genes might influence secretion of capsule material.

**Fig 2:**
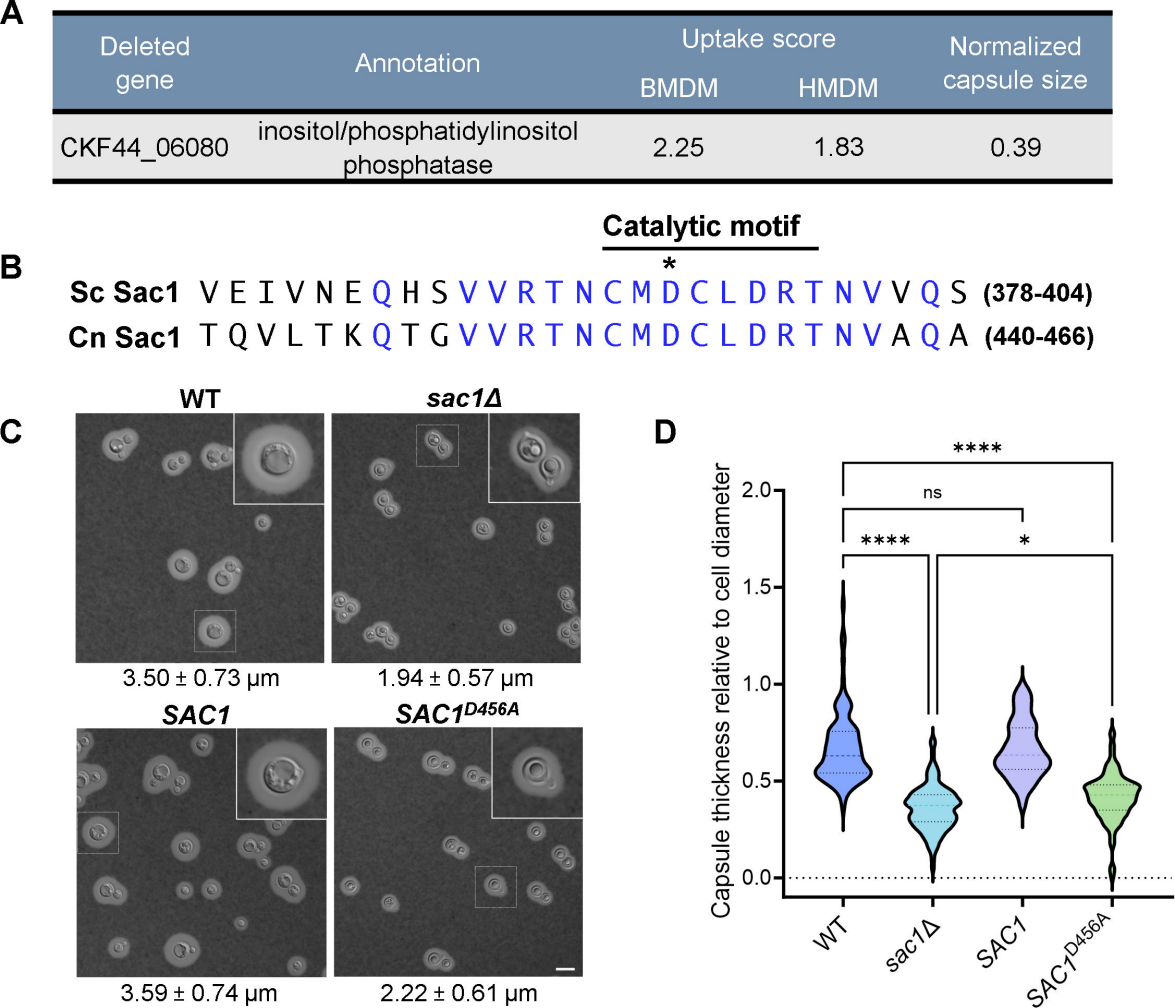
Cells lacking active Sac1 show increased uptake by phagocytes and smaller capsules. (**A**) Uptake and capsule screen results for cells lacking CKF44_06080. (**B**) Alignment of *C. neoformans* and *S. cerevisiae* sequences that include the Sac1 catalytic motif. Blue, identical residues; asterisk, residue mutated to alanine in the *SAC1^D456A^* strain. (**C**) India ink negative stain of the indicated strains grown for 24 h in Dulbecco's modified Eagle medium (DMEM) at 37°C with 5% CO_2_. Capsule thickness in micrometer (mean ± SD) is noted below each panel. All primary images are at the same magnification; scale bar, 10 µm. For this and other microscopy figures, any region demarcated by a small square is enlarged in the inset at upper right. (**D**) Distribution of normalized capsule thicknesses for the indicated strains. At least 96 cells were measured for each strain as described in Materials and Methods. *, *P* < 0.0332; ****, *P* < 0.0001 by two-way analysis of variance with Tukey’s multiple comparison test; ns, not significant.

Among the eight mutants likely impaired in secretion, we particularly noticed one strain that showed increased phagocytosis by both BMDM and HMDM and a 61% reduction in capsule thickness compared to WT cells (Fig. 2A). Its missing sequence, *CKF44_06080*, encodes a protein with 38% amino acid identity to the phosphatidylinositol phosphatase Sac1 of *Saccharomyces cerevisiae,* including 85% identity across a 20-amino acid region that encodes a conserved CX_5_R(RT) motif necessary for catalytic function (33). Interestingly, despite this similarity, ectopic overexpression of *S. cerevisiae* Sac1 did not restore the capsule defects of *sac1*Δ cells (Fig. S1).

The phenotypes of *sac1*Δ cells, together with the unique biology of the capsule and its importance as a virulence factor, led us to further investigate the role of Sac1 in *C. neoformans*. To pursue these studies, we first engineered a new Sac1 deletion strain (*sac1*Δ) and complemented the mutant at the endogenous site (*SAC1*). We also identified the conserved Sac1 catalytic motif by homology to *S. cerevisiae* Sac1 and inactivated it by replacing an aspartic acid residue with alanine (*SAC1^456A^*, Fig. 2B; Fig. S2) (34–36). Using these strains, we confirmed that loss of Sac1 led to thinner capsules, a phenotype which was maintained after normalization to the smaller diameters of the *sac1*Δ cells (Fig. 2C and D). These traits were phenocopied by the inactivated strain, showing that the phosphatase function of Sac1 is essential for capsule production, while complementation restored WT phenotypes (Fig. 2C and D). While imaging, we also observed striking round structures in the *sac1* mutant cells (Fig. 2C, right panels). These were distinguished from typical organelles, like the vacuoles visible in WT and *SAC1* cells, by their circular shape, defined edge, and striking refractility and only occurred in *sac1* mutants grown in host-like conditions (here defined as Dulbecco's modified Eagle medium [DMEM], 37°C, 5% CO_2_). These structures will be further discussed below.

### Sac1 is required for cryptococcal virulence

**Fig 3:**
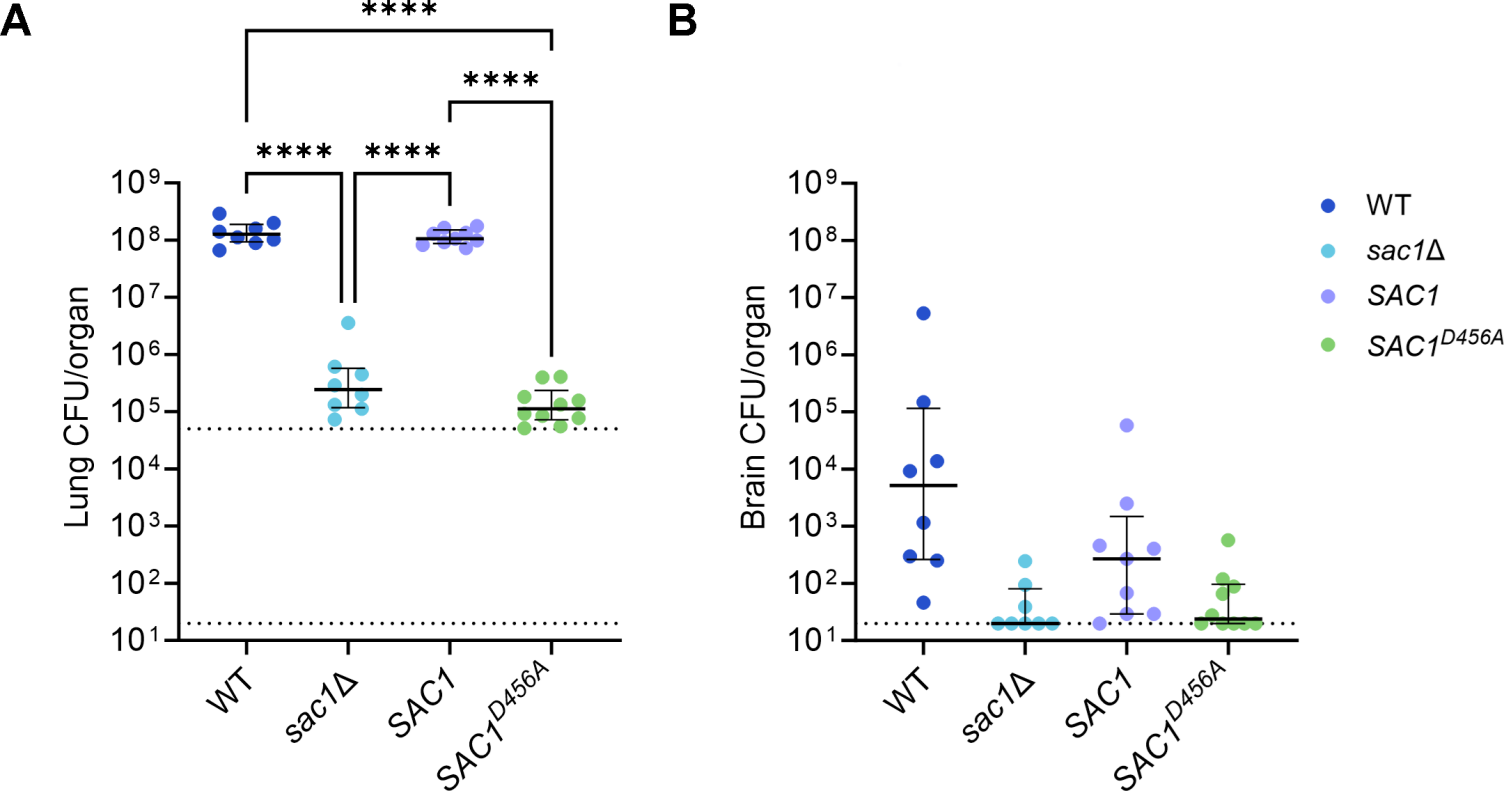
Sac1 is important for virulence in a mouse infection model. (**A**) Lung and (**B**) brain burden of C57BL/6 mice (groups of 8–10) sacrificed 14 days after intranasal infection with 5 × 10^4^ fungal cells (upper dotted line). Plots show median (bold black line) with interquartile range. Lower dotted line, limit of detection. Each symbol represents one mouse. ****, *P* < 0.0001; comparison with no stars shown are not significant. Significance was calculated using two-way analysis of variance with Tukey’s multiple comparison test.

Variations in both uptake and capsule size are associated with changes in pathogenicity (9, 37–43), so we suspected that *sac*1 mutants would have impaired virulence. To test this, we infected mice intranasally and measured organ burden at 14 days post-infection. Notably, while *sac1*Δ cells were recovered from the lungs, they remained close to the level of the original inoculum. In sharp contrast, the lung burden of WT and *SAC1*-infected animals increased roughly 2,000-fold in this period (Fig. 3A). Cells were recovered from the brain in only a minority of mutant infections, although all WT infected animals had detectable brain burden by this time point (Fig. 3B). We observed similar results in a time course study, which demonstrated gradual accumulation of cryptococci in the lung up to 18 days post-infection (Fig. S3A) and impaired dissemination to the brain (Fig. S3B). These results motivated us to define the mechanism(s) by which Sac1 influences virulence.

### Sac1 controls substrate distribution and sensitivity to stressors

**Fig 4:**
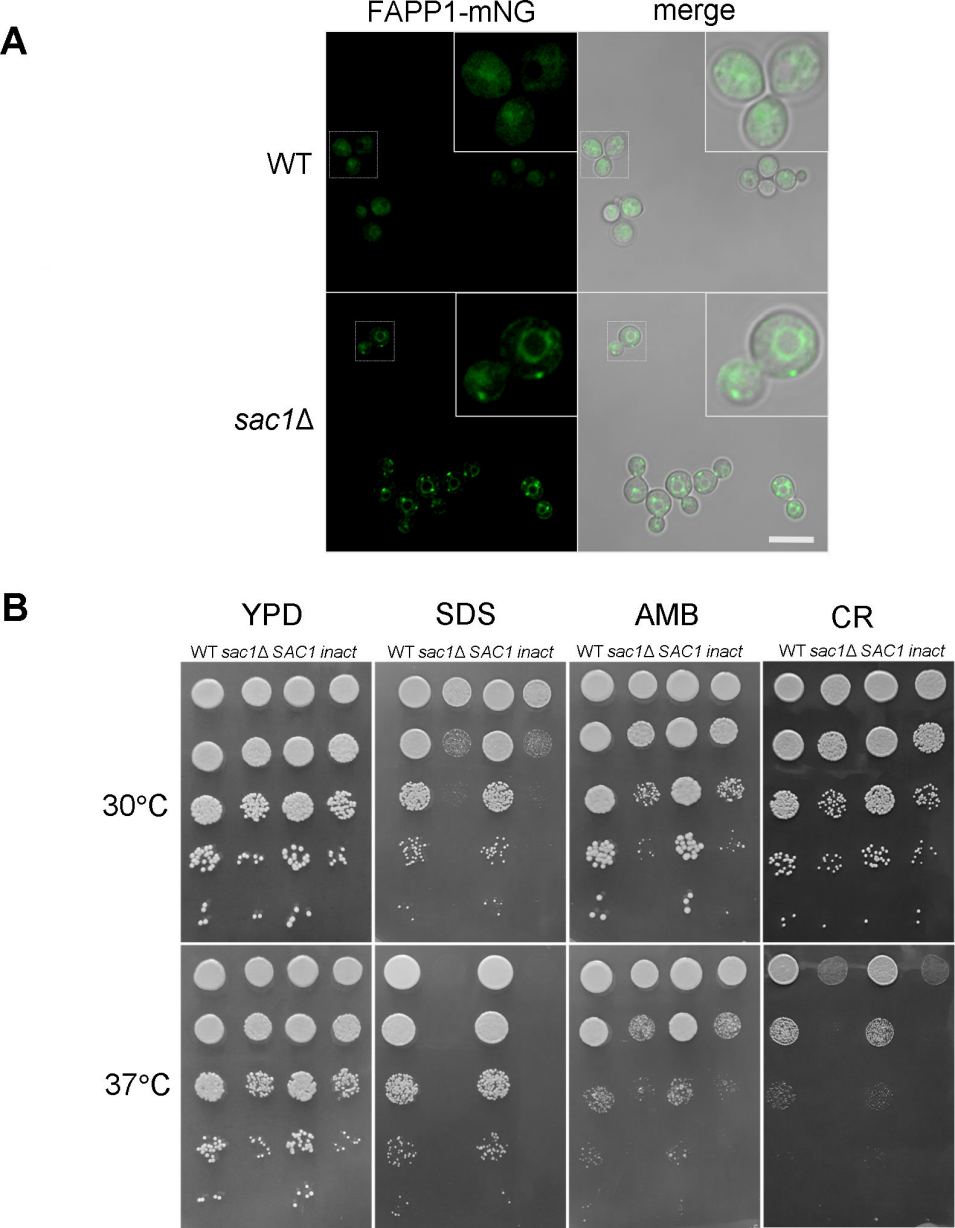
Sac1 regulates substrate distribution and stress sensitivity. (**A**) FAPP1-mNeonGreen (mNG) localization in cells grown in yeast extract-peptone-dextrose (YPD) at 30°C. All primary images are at the same magnification; scale bar, 10 µm. (**B**) Serial 10-fold dilutions of the indicated strains plated on YPD alone or containing 0.01% SDS (SDS), 0.05% Congo Red (CR), or 2 µg/mL amphotericin B (AMB), incubated at 30 and 37°C. *inact* = *SAC1^D456A^*.

Sac1 has not been characterized in *C. neoformans*. To test its influence on turnover of intracellular PI4P, we engineered strains to express an mNeonGreen-tagged peptide that binds PI4P (FAPP1) and has been used to assess its localization (44). We then examined the abundance and localization of this fluorescent peptide (Fig. 4A). Overall, the FAPP1 signal was brighter in *sac1*Δ cells than in WT, a trend supported by flow cytometry (Fig. S4). This is consistent with a role for Sac1 as a PI4P phosphatase, as loss of this protein would lead to an increase in its substrate. We also noted distinctive bright perinuclear rings in the *sac1*Δ cells (especially in rich medium), which were more diffuse in WT cells. These correspond to an endoplasmic reticulum distribution (45) and suggest aberrant accumulation of PI4P in the early secretory pathway. Finally, the mutant strain exhibited intense puncta, absent from WT cells, which are consistent with PI4P accumulation in the yeast Golgi (46).

Secretion is a vital cellular process, so its perturbation could have multiple deleterious effects on the cell, including effects on the plasma membrane and cell wall. To examine this, we tested strain growth in various stressful conditions. Although both *sac1* mutants grow slightly slower than WT or complemented cells even on rich medium, they showed much worse growth on media containing membrane stressors (the detergent SDS and the sterol-binding antifungal amphotericin B) or the cell wall stressor Congo Red (Fig. 4B). These defects were exacerbated when cells were grown at 37°C. In all of our assays, the complemented strain phenocopied WT and the inactivated construct phenocopied the deletion mutant.

### Sac1 regulates cellular lipids during the response to host-like conditions

**Fig 5:**
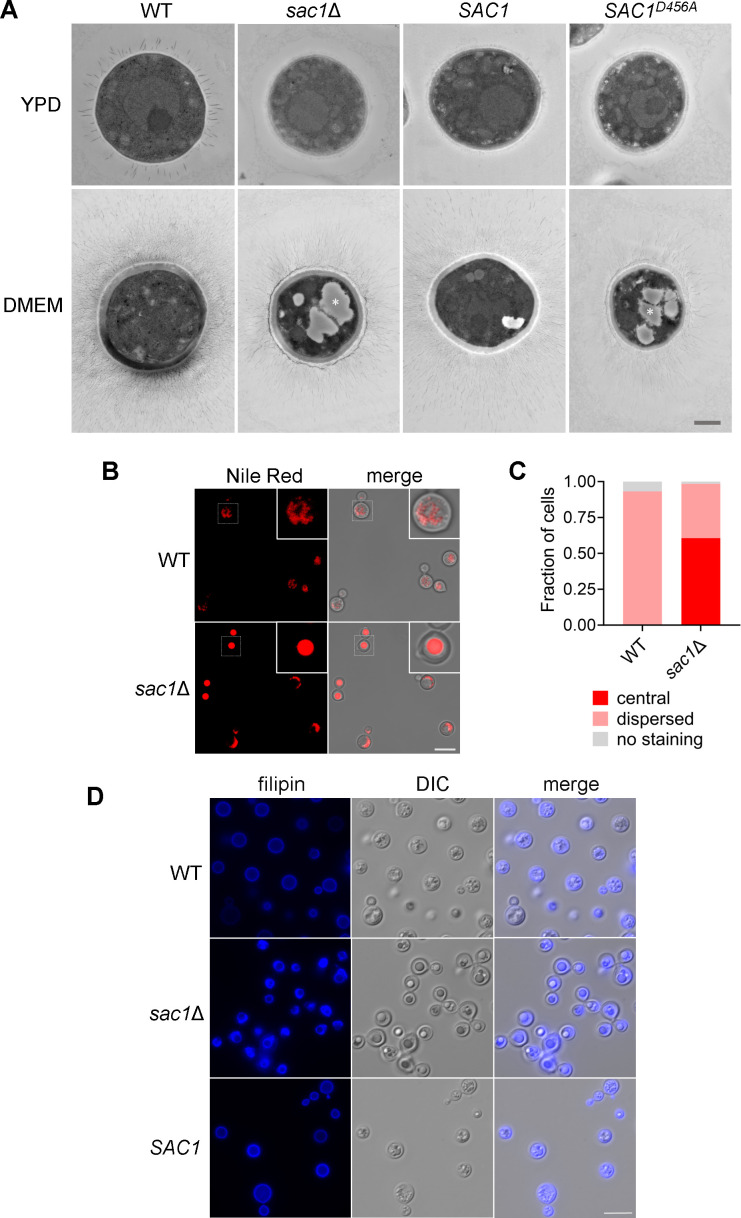
Sac1 is essential for proper lipid trafficking in host-like conditions. (**A**) Transmission electron micrographs of cells grown in the indicated medium. White asterisks, example lipid droplet structures, likely distorted during sample prep; scale bar, 1 µm. (**B**) Nile Red stain of the indicated strains with (**C**) quantification of the observed staining pattern. (**D**) Filipin staining of cells grown in DMEM. Yeast extract-peptone-dextrose (YPD) grown cells in panel A were grown overnight at 30°C, all other cells were grown for 24 h in DMEM at 37°C with 5% CO_2_. Scale bar, 10 µm. All images within each experiment are shown at the same magnification.

We sought to explain the capsule defect and stress sensitivities of *sac1* mutants based on the cellular consequences of perturbed PI4P turnover. In other eukaryotes, PI4P is important for establishing the lipid composition of secretory vesicles (47) and its turnover is critical for the progression of post-Golgi secretory traffic (48). We therefore examined classes of molecules, including lipids, proteins, and capsule polysaccharides, that are directly or indirectly influenced by this step of secretion. As noted above, we had observed formation of large internal structures in *sac1* mutant cells grown under host-like conditions (Fig. 2C), which were absent from WT and complemented cells. Because of their shape and refractile appearance in light micrographs, we suspected they were at least in part composed of lipid. Consistent with this suggestion, they appeared electron lucent by transmission electron microscopy (Fig. 5A, asterisks). We hypothesized that these structures result from aberrant lipid handling within *sac1* mutant cells that is induced by the stress of host-like conditions, since they are absent from mutant cells grown in rich medium (Fig. 5A). Based on this idea, and our results below, we termed them Sac1 lipid-accumulating bodies (SLBs).

SLBs resemble previously characterized “supersized” lipid droplets identified in *S. cerevisiae* (49). Lipid droplets are neutral lipid-containing structures that represent essential cellular energy reserves; these increase in abundance when cryptococcal cells are grown in host-like conditions (50). When we stained for neutral lipids using Nile Red, WT cryptococcal cells showed dispersed punctate signal, while *sac1*Δ cells showed concentrated central staining, frequently colocalized with SLBs (Fig. 5B and C) and thus supporting the presence of neutral lipids within them.

We recently demonstrated that mislocalization of the important fungal lipid ergosterol leads to accumulation of lipid droplets (50). We had also noted that *sac1* mutants were more sensitive than WT cells to amphotericin B, which binds ergosterol (Fig. 4B). Consequently, we wondered whether ergosterol localization in *sac1*Δ cells was perturbed. When we stained WT cells grown in host-like conditions with filipin, which binds non-esterified sterols, we saw the typical ring staining expected for a component of the plasma membrane (Fig. 5D). By contrast, mutant cells exhibited increased internal staining, particularly bordering the SLBs (see Discussion). When we tested filipin and Nile Red together, both stains localized to SLBs in the *sac1* mutants, with a ring of filipin surrounding the Nile Red stain (Fig. S5). In contrast, WT cells showed peripheral filipin staining and Nile Red staining dispersed throughout the cell.

**Fig 6:**
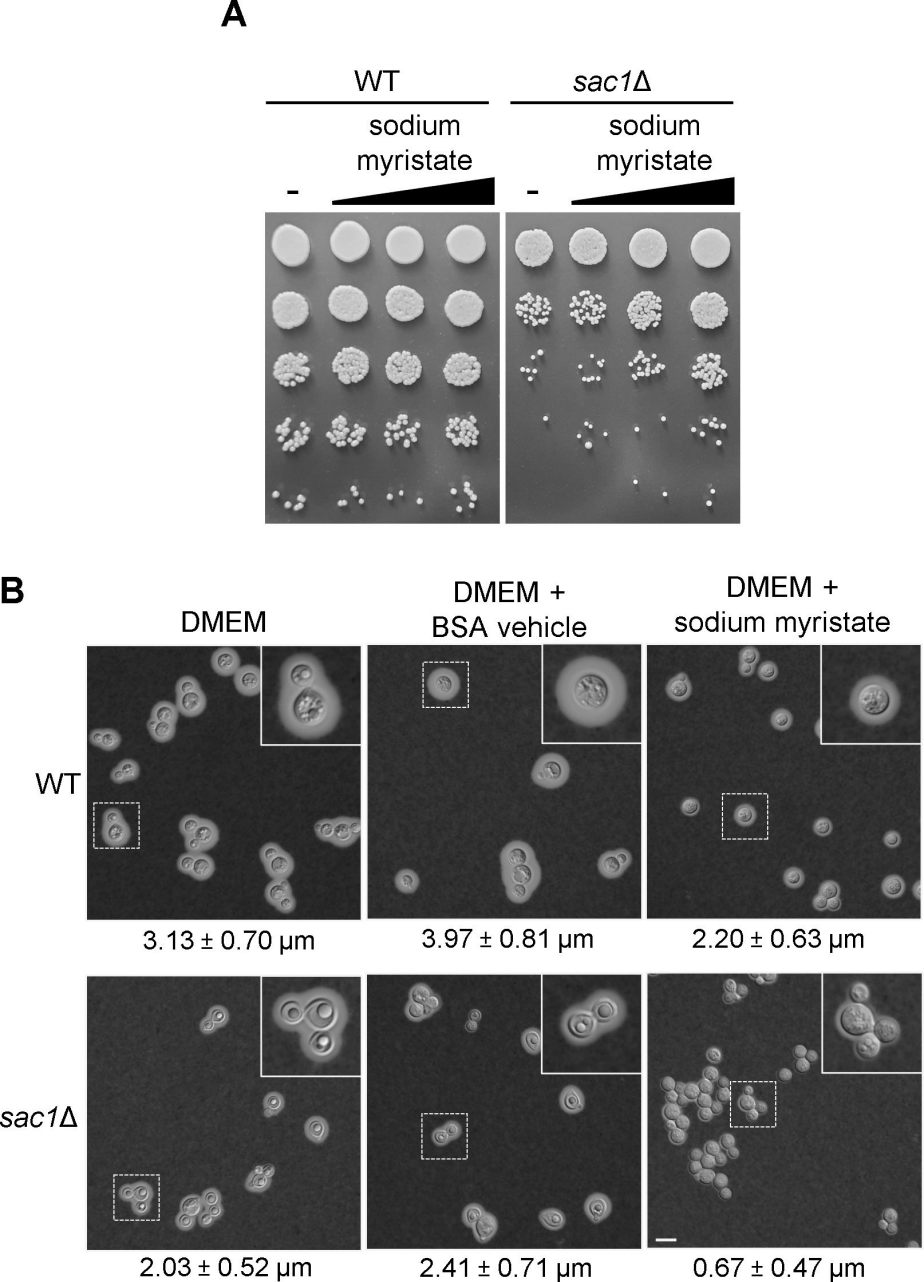
Supplementation with exogenous fatty acid partially restores *sac1*Δ growth and morphology but not capsule biosynthesis. (A) Serial 10-fold dilutions of WT and *sac1*Δ cells grown for 24 h (at 37°C with 5% CO_2_) in DMEM supplemented with BSA alone (-), or with 8, 40, or 200 μM myristic acid conjugated to BSA (increasing amounts indicated by the triangle). (B) India ink negative stain of WT and *sac1*Δ cells grown in DMEM at 37°C with 5% CO_2_, supplemented as indicated with BSA alone or 200 μM myristic acid conjugated to BSA. Capsule thickness in micrometer (mean ± SD) is noted below each panel. All primary images are to the same magnification; scale bar, 10 μm.

In testing cell growth, we fortuitously discovered that supplementation of host-like conditions (DMEM, 37°C, 5% CO_2_) with various exogenous fatty acids partially restored the growth of *sac1*Δ cells (Fig. 6; Fig. S6); this effect was dose-dependent (shown for myristate in Fig. 6A). This supplementation also prevented the formation of SLBs (Fig. 6B, bottom right panel). When we visualized capsule in myristate-supplemented cells, we made several observations. First, the addition of the bovine serum albumin (BSA) vehicle alone led to a slight capsule size increase, of similar magnitude, in both cell types. Second, myristate supplementation yielded significant capsule reduction in both strains, which was more dramatic in the mutant (Fig. 6B). Lipid supplementation thus restores growth and prevents SLB formation in *sac1*Δ cells but does not rescue capsule size (see Discussion).

### Sac1 impacts protein secretion and localization

**Fig 7:**
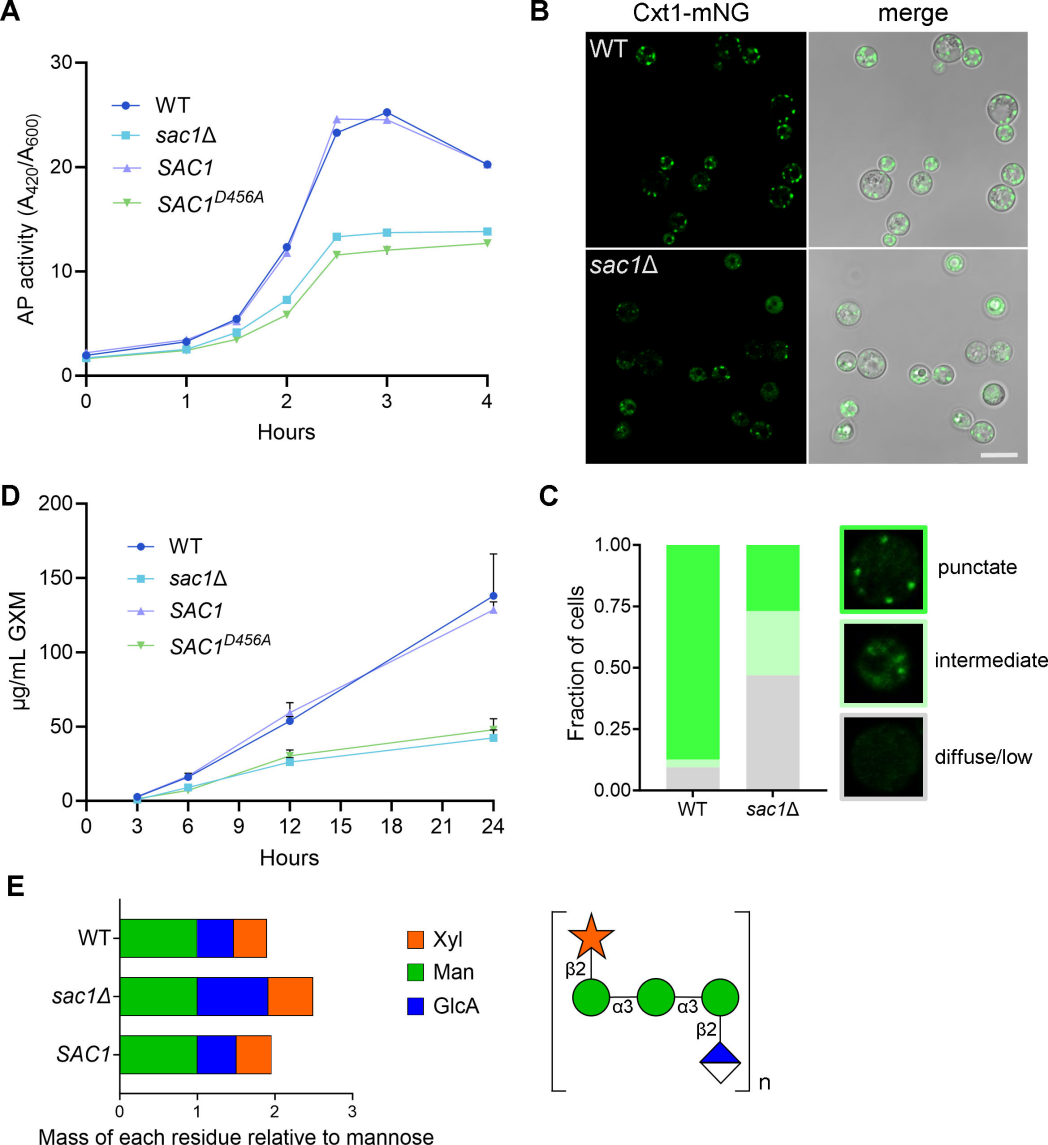
Sac1 controls the secretory pathway to modulate capsule size and composition. (A) Secreted acid phosphatase activity, normalized to cell number, over time after a shift to phosphate-depleted medium. (B) Confocal imaging of Cxt1-mNeonGreen (mNG) in the indicated strain backgrounds with (C) quantification of observed staining patterns. Punctate: three or more puncta present; intermediate: puncta and diffuse signal; diffuse/low: diffuse signal or <3 puncta. Images are to the same scale; scale bar, 10 μm. (D) Shed glucuronoxylomannan (GXM) quantified by enzyme-linked immunosorbent assay (ELISA). For both (A) and (D), mean ± SD for one representative experiment of three biological replicate studies is shown. (E) Left, GXM composition; right, subunit structure of GXM.

Proteins are critical secretory cargo, so it is likely that accumulation of PI4P in the secretory pathway would affect their transport and secretion. To assess this, we measured protein secretion directly, using inducible acid phosphatase production as a proxy for general protein secretion (24). Upon entry into phosphate-depleted conditions, WT, *SAC1*, and both *sac1* mutants began producing detectable levels of acid phosphatase within 1 h. While the general patterns of secretion were similar, the mutants produced less acid phosphatase on a per cell basis throughout the time course (Fig. 7A), suggesting an overall reduction in protein secretion.

Perturbations in PI4P levels may affect not only proteins secreted outside the cell, but also those resident in the secretory system, including multiple capsule biosynthetic proteins in the Golgi apparatus. We hypothesized that the loss of PI4P turnover could result in failure to correctly localize these enzymes and thereby explain the altered capsule of *sac1* mutants. To investigate this question, we localized the best-characterized capsule biosynthetic enzyme, Cxt1 (51), in *sac1*Δ and WT cells grown in host-like conditions. WT cells displayed clear puncta of Cxt1-mNeonGreen, consistent with the expected Golgi localization of this protein. In contrast, staining in *sac1*Δ cells was heterogenous, with various cell populations displaying punctate, diffuse, or little to no fluorescence (Fig. 7B and C).

### Sac1 affects capsule secretion and composition

Capsule glycans constitute a class of secretory cargo which is unique to *C. neoformans*. The predominant capsule polysaccharide, glucuronoxylomannan (GXM), traverses the secretory pathway prior to extracellular release (24). We hypothesized that GXM secretion, like protein secretion, is reduced in *sac1*Δ cells, causing their thin capsules. To test this, we quantified GXM secretion at intervals after transition to host-like conditions. Importantly, WT and *sac1* mutants began to produce detectable levels of GXM concurrently, showing that the mutants respond normally to the environmental signals that trigger capsule production (Fig. 7D). However, the overall amount of GXM they secreted was markedly reduced.

Reduced GXM secretion could certainly explain the small capsules of *sac1* mutant cells. Another reason for altered capsule could be mislocalization of glycosyltransferases (such as we observed with Cxt1), since compositional differences in the polysaccharide also affect capsule size and architecture (8). To investigate this, we analyzed the composition of GXM purified from WT, *sac1*Δ, and *SAC1* cells. Compared to the WT and *SAC1* controls, *sac1*Δ GXM showed a substantial (~2-fold) increase in the relative proportion of glucuronic acid, as well as a modest increase in xylose (Fig. 7E). These results were supported by linkage analysis, in which we observed a reduction in 3-linked mannopyrannosyl residues as a fraction of the total (Table S3), indicating fewer unmodified backbone mannose residues.

## DISCUSSION

How *C. neoformans* interacts with host cells is a critical determinant of infection. In this study, we screened 4,692 cryptococcal deletion strains and identified 93 that demonstrated altered uptake by both mouse and human primary phagocytes. GO analysis showed that this set was enriched in sequences that encode proteins with roles in cytoskeletal interactions, protein transport, and organization of the cell wall and capsule. This is consistent with our understanding that the outer layers of the cell and secreted factors modulate interactions with host cells. We also identified strains with defects in only one host system. Why the species differ in this regard could be a fruitful area for future research.

Capsule is a critical factor in the interaction between *C. neoformans* and host cells, but the mechanisms of its synthesis and transport remain largely unknown. To approach this question, we performed a secondary screen for altered capsule thickness, which yielded 131 mutants. For comparison, a previous screen of 1,201 deletion strains, using colony morphology as the primary screen, yielded 16 capsule-related genes (19). Furthermore, our hits represent approximately one-third of the original set of uptake mutants that we tested. This shows that our strategy of using these mutants as the starting population to screen for capsule defects was highly successful in enriching for capsule phenotypes. Notably, 42 of the hits are annotated as encoding hypothetical proteins (52), which may be subjects for future investigation. The others were enriched for GO terms related to cell wall and capsule organization, which validated our methods, and for terms involving transporter activity, intracellular transport, and phosphatidylinositol binding. As our results with Sac1 demonstrate, the study of such processes, which are not directly related to capsule production, may still yield insight into capsule biosynthesis.

Interestingly, over half of our capsule screen hits were hypercapsular strains. This highlights an important strength of our image-based technique, as prior methods (including colony morphology, antibody blotting, or volume-based methods) identify strains that lack capsule more readily than hypercapsular cells (53). That said, image-based screens may also have weaknesses. For example, mutants with general morphological defects may register as false positives, as cells that fail to divide might appear to be one large cell with a capsule defect. Further, this method cannot identify mutants that are completely acapsular, as they will not bind antibody.

Of the 45 gene products identified as hits in all three of our screens, the largest subset of the uncharacterized hits was predicted to be involved in the secretory pathway. We focused on one of these hits, Sac1, a PI4P phosphatase encoded by *CKF44_06080*. The *CNAG_06080* gene (which encodes Sac1 in the closely related reference strain H99) was previously identified as a site where random integration of a drug marker yielded an attenuated *C. neoformans* strain (54). Like our KN99 mutant, this strain showed reduced virulence, although direct comparison is limited because the mutations, mouse models, and experimental designs differ. Other investigators had proposed that this gene was essential, because they were unable to delete it (55). We expect this difference from our findings was due to the challenge of working with cells that have significant defects, although it could also reflect different strain backgrounds (H99S vs KN99) (56, 57).

By expressing and localizing a fluorescent PI4P-binding peptide (44, 58, 59), we supported a role for Sac1 in PI4P turnover in *C. neoformans*. Although we did not test this activity directly, based on homology to the well-characterized *S. cerevisiae* Sac1 protein, conservation of Sac1 function throughout eukaryotes, and our binding data, we believe PI4P is the main substrate of Sac1 in *C. neoformans*. It is interesting that despite its high amino acid similarity to the *S. cerevisiae* Sac1 protein, overexpression of the latter did not rescue the defects of *C. neoformans sac1*Δ cells; this result may warrant further experimentation.

When s*ac1* mutants are grown in host-like medium, they develop large structures which we termed SLBs. The interiors of SLBs stain with Nile Red, which binds neutral lipids, and the edges stain with the sterol-binding dye filipin. SLBS thus share certain features with lipid droplets, which are composed of a neutral lipid core of mainly triacylglycerols and sterol esters surrounded by a phospholipid monolayer (60). More specifically, SLBs resemble “supersized” lipid droplets, which form in model yeast strains defective in phospholipid metabolism (49, 61). This phenomenon has also been observed in *C. neoformans* following treatment with the antidepressant sertraline (62). However, our filipin staining suggests the presence of ergosterol or sterol intermediates (63) on the perimeter of the SLBs, which has not been reported for regular or supersized lipid droplets; accordingly, we have not adopted this terminology.

Ergosterol is distributed in a steeply increasing gradient from the endoplasmic reticulum (ER) to the plasma membrane (64). One model proposes that PI4P turnover by Sac1 maintains this gradient (65). If this mechanism occurs in *C. neoformans,* loss of PI4P turnover would likely result in the inability to shuttle ergosterol up its concentration gradient with consequent accumulation in the ER. Another model suggests that the ergosterol gradient depends on membrane phospholipid composition (64). In either case, loss of Sac1 could perturb lipid transport and homeostasis and lead to mislocalization of ergosterol.

PI4P is a key signaling lipid, that serves as a pro-secretory marker and modulates the exit of secretory traffic from the *trans-*Golgi (27, 47, 66). We propose that the lack of PI4P turnover in *sac1* mutants leads to its accumulation in the early portions of the cryptococcal secretory system. This would be consistent with our observations of reduced protein secretion and mislocalization of the Golgi-resident protein Cxt1 in *sac1*Δ cells. The Golgi serves as a central hub for trafficking of both proteins and lipids, and in doing so plays a key role in membrane trafficking and lipid metabolism (67). We hypothesize that SLAB formation in *sac1* mutants represents the failure of lipid trafficking under stressful, nutrient-limited host-like conditions, leading to the accumulation of lipid cargo and/or lipid metabolic precursors. SLAB formation and the poor growth of *sac1*Δ cells is prevented by fatty acid supplementation, which somehow bypasses the defect caused by PI4P accumulation and allows the cells to grow normally. How this occurs will be an interesting question for future research. Importantly, while fatty acid supplementation rescues SLAB formation in *sac1*Δ cells, capsule production is not restored. This key finding shows that the adverse effects of Sac1 loss occur in divergent pathways.

Capsule material is exported in secretory vesicles (24), so defects in anterograde traffic would also impair this process. Beyond reduced GXM secretion, which is consistent with this expectation, we observed alterations in GXM composition: the mannose backbone had a large increase in glucuronic acid substitution and a slight increase in xylose. We hypothesize that altered localization of capsule biosynthetic enzymes changes their access to nascent capsule material and precursor molecules. It is not known whether capsule polysaccharides traverse the cell as subunits or extended polymers (68, 69), but in either case, increased exposure to biosynthetic enzymes could result in compositional changes such as we observed. Both the reduction of capsule size and changes in composition in *sac1* mutants likely contribute to their increased uptake by host phagocytes.

In this study, we identified *C. neoformans* mutants impaired in host interactions with primary cells and assessed the hits for capsule defects. This strategy greatly increased the number of sequences known to influence capsule production, including many previously uncharacterized genes. Analysis of these genes and the corresponding mutants will help us understand the processes required for synthesis of this central virulence factor, as exemplified in this work on Sac1. Importantly, two-thirds of our original uptake hits did not have altered capsule thickness, implicating additional mechanisms in this fundamental host-pathogen interaction. Further studies will be required to determine what leads to the altered uptake of these strains, which may include changes to the cell wall or surface mannoproteins (31) or structural alterations in the capsule that do not impact its thickness.

## MATERIALS AND METHODS

### Strain construction, validation, and growth

*sac1Δ*, *SAC1* complement, and *SAC1^D456A^* strains were made with split-marker biolistic transformation (70) in KN99α. Cxt1 was tagged with mNEONGREEN (pBHM2404, Addgene #173441, a gift from Hiten Madhani) using CRISPR with short homology-directed repair (71). FAPP1-mNeonGreen (from pRS406-PHO5-GFP-hFAPP1_PH_domain, Addgene #58723, a gift from Tim Levine) and *S. cerevisiae SAC1* strains were similarly inserted in the Safe Haven 2 region (71, 72).

Transformants were validated by antifungal resistance, PCR, and/or whole genome sequencing. Expression of *S. cerevisiae* S288C *SAC1* was confirmed by extracting total RNA with TRIzol Reagent, synthesizing cDNA using SuperScript III (Thermo Fisher, #18080400), and PCR amplifying a unique region of the gene (Fig. S1B).

For all assays except screening, *C. neoformans* strains were grown on yeast extract-peptone-dextrose (YPD) plates (2 days, 30°C), and single colonies were inoculated into YPD and grown overnight (30°C, shaking). For capsule inductions, cells were washed in phosphate-buffered saline (PBS), inoculated in DMEM at 10^6^ cells/mL, and grown for 16–24 h (static, 37°C, 5% CO_2_). For screening, cells were cultured from glycerol stocks in 96-well plates, grown overnight in YPD, and then sub-cultured and grown 24 h in YPD. Detailed methods, including stress media and lipid supplementation, are in the supplemental material.

### Screening

For uptake screening, C57BL/6J mouse BMDMs were harvested as in reference (73) and differentiated for 9 days before isolation. HMDMs were from anonymous donors. Peripheral blood mononuclear cells (PBMCs) were isolated by Ficoll-Paque centrifugation and differentiated for 6 days. The screen assay was modified from reference (23). Briefly, opsonized Lucifer Yellow-stained fungi were added to host cells at a multiplicity of infection of 20 (BMDM) or 30 (HMDM) and the cultures were co-incubated for 1 h and washed. Adherent cells were stained with Calcofluor White; wells were washed, fixed, and permeabilized; and host cell nuclei were stained with propidium iodide before imaging on a BioTek Cytation 3. The uptake index (internalized cells per macrophage) was corrected for the number of fungal cells added and then normalized to the plate median (first round) or KN99 value (later rounds).

For capsule screening, strains identified as hits in the first round BMDM uptake screen were assessed for capsule size differences using slight modifications of our previous method (32). For this, cells were induced as above, capsules were stained with anti-GXM mAb-302-AF488, and cell walls were stained with Calcofluor White. Images were collected as above, and capsule thickness measured as in reference (33).

Further details of methods and hit selection for both screens are in the supplemental material.

### Virulence studies

Six- to 8-week-old female C57BL/6 mice (The Jackson Laboratory) were anesthetized by subcutaneous injection of 1.20  mg ketamine and 0.24 mg xylazine in 110 µL sterile PBS. For 14-day infections, mice were intranasally infected with 5  ×  10^4^ cryptococcal cells. For time course infection, mice were infected with 1.25 × 10^4^ cryptococcal cells and sacrificed at 6, 12, and 18 days post-infection. For both infections, the lungs and brains were harvested, homogenized, and plated on YPD agar to assess fungal organ burden.

### Assays

Acid phosphatase secretion was measured as in reference (24). In addition, 10^7^ cells/mL cultures were grown in MM-KCl (0.5% KCl, 15 mM glucose, 10 mM MgSO4⋅7H2O, 13 mM glycine, 3.0 µM thiamine). Supernatants were sampled, incubated with substrate buffer (2.5 mM para-nitrophenylphosphate in 50 mM acetate buffer, pH 4.0) at 37°C for 5 min, and stopped with 800 µL saturated Na_2_CO_3_. Acid phosphatase secretion was quantified as A_420_/OD_600_.

GXM enzyme linked immunosorbent assays (ELISAs) were performed as in reference (51). Cultures were induced in DMEM,and the supernatants filtered (0.2 µm). Flat-bottom Immulon 1B plates (Thermo Scientific) were coated with 1 µg/mL anti-GXM antibodies 339 and F12D2γ, washed with PBS with 0.5% TWEEN 20 (PBST), and blocked for 90 min with 1% BSA in PBST. Filtrates were diluted in PBST and incubated on the plate for 90 min. The plate was washed three times, probed with horseradish peroxidase-labeled mAb 339/F12D2γ, incubated with KPL TMB Microwell Peroxidase Substrate (SeraCare), developed, and quantified using A_450_.

### Imaging

For capsule visualization, cells were induced as above, mixed with India ink in PBS (1:2, vol/vol), and imaged by light microscopy. Capsules of at least 96 cells from randomly chosen fields were measured using ImageJ. Nile Red and filipin staining was performed as in reference (50), and viewed on a ZEISS Axio Imager M2 fluorescence microscope. FAPP1-mNEONGREEN and Cxt1-mNEONGREEN stains were imaged with a Zeiss LSM880 confocal microscope. Morphological classification of Cxt1-mNeonGreen and Nile Red staining was performed on at least 94 cells from randomly selected fields. Electron microscopy was as in reference (50) with one change detailed in Supplement.

### GXM analysis

GXM was isolated using precipitation with hexadecyltrimethylammonium bromide as in reference (74). Isolated material was hydrolyzed with trifluoroacetic acid (3 h, 100°C) and composition assessed on a Dionex ICS-6000 instrument. Linkage analysis was performed by the Complex Carbohydrate Research Center [method modified from reference (75)].

## SUPPLEMENTAL MATERIAL

Supplemental material mbio.01496-24-s0001.pdf Supplemental methods, table captions, and figures.

Table S1 mbio.01496-24-s0002.xlsx Uptake screen results.

Table S2 mbio.01496-24-s0003.xlsx Capsule screen results.

Table S3 mbio.01496-24-s0004.docx Linkage analysis.

ASM does not own the copyrights to Supplemental Material that may be linked to, or accessed through, an article. The authors have granted ASM a non-exclusive, world-wide license to publish the Supplemental Material files. Please contact the corresponding author directly for reuse.
