# Supplementary material for: Sac1 links phosphoinositide turnover to cryptococcal virulence
Source: mBio. 2024 Jul 2;15(8):e01496-24. doi: 10.1128/mbio.01496-24 (PMC11323556; doi:10.1128/mbio.01496-24)
Supplement: Supplemental material — Supplemental methods, table captions, and figures. [file mbio.01496-24-s0001.pdf]

SUPPLEMENTAL MATERIAL FOR GAYLORD ET AL

Supplemental Methods

BMDM preparation

BMDMs were isolated as in (73) from 9-week old C57BL/6J mice and frozen in 90% FBS, 10% DMSO at 10^7 cells/mL. Thawed cells were differentiated for 9 days in RPMI-1640 with L-glutamine (Sigma) with 30% L-cell supernatant and 20% heat-inactivated fetal bovine serum (Gibco), with regular media changes. BMDM were resuspended in 10 mL ice cold PBS and isolated using 2.5 µg anti-mouse F4/80 biotin (clone BM8, eBiosciences) plus 0.5 µg mouse BD FC-Block (BD Pharmingen) with anti-biotin microbeads (Miltenyi Biotec) and a MACS separation column (Miltenyi Biotec).

Human macrophage preparation

Leukoreduction system (LRS) chambers were obtained from anonymous donors and human peripheral blood mononuclear cells (PMBC) were isolated by Ficoll-Paque centrifugation (Lymphoprep; Axis-Shield). 5x10^7 PMBC were allowed to adhere to a treated culture dish (Corning) for 2 hours at 37°C with 5% CO_2. Detached cells were removed, and adherent cells were maintained in RPMI-1640 differentiation media containing L-glutamine (Sigma), 10% fetal bovine serum (Sigma), 1% penicillin streptomycin (Fisher), 1% sodium pyruvate (Corning), 50ng/mL human recombinant M-CSF (Fisher), and 1% MEM non-essential amino acids (Corning). Medium was replaced every two days and adherent cells were harvested on day 6 using Cell Stripper (Corning).

Uptake screen

Assays were performed using a modification of our prior method (23). Host cells were seeded in flat clear bottom black polystyrene TC-treated 96-well plates (Corning) at a density of 10^5 cells/well and incubated for one day at 37°C with 5% CO_2. *C. neoformans* strains were cultured from frozen stocks in glycerol/Yeast Extract-Peptone-Dextrose medium (YPD) in 96 well format for 18-24 h at 30°C with shaking (800 rpm) (Southwest Science, SBT1500-H). At least 2 µL of cells were then sub-cultured in 100 µL YPD and grown for 18-24 h as above. After sub-culture, cells were sedimented (3000 g, 2 min, RT), washed twice with 200 µL of PBS, washed once with 200 µL McIlvaine's Buffer [pH 6], and resuspended in 80 µL of the same. Cells were then stained with 5 mg/mL Lucifer Yellow (30 min, RT, 650 rpm) and resuspended in 100 µL of PBS. To account for variation in growth rates of *C. neoformans* mutants, OD_{600} was used to roughly standardize cell number and maintain results in the linear range of the assay. To do this, individual mutants were categorized as having high, medium,

or low growth based on the OD_{600} after resuspension in PBS and the average value of each group was used to prepare wells with approximately 10^7 fungal cells/mL in PBS. Fungal cells were then opsonized with 10% by volume C57Bl6/J mouse serum (for BMDM) or human serum from male AB plasma (for HMDM, Sigma) for 30 minutes at 37°C at 250 rpm. Opsonized cells were then added to wells containing RPMI and macrophages at a multiplicity of infection of 20 (BMDM) or 30 (HMDM). Cells were co-incubated for one hour at 37°C with 5% CO_2 to allow uptake and washed. This and the following washes were performed using a Biotek ELx405 Microplate Washer (2 washes, PBS). Adherent fungal cells were stained with 150 µL of 5 µg/mL Calcofluor White (Sigma) (15 min, 4°C, static). Samples were then washed, fixed with 4% formaldehyde (10 min, RT), washed, permeabilized with 1 mg/mL saponin (20 min, RT), washed, and stained with 50 µg/mL propidium iodide (15 min, RT). Imaging was performed on a BioTek Cytation 3 imager collecting 12 images per well in a 4x3 grid pattern.

A separate plate of each *C. neoformans* strain alone was counted to allow normalization for the number of cells of each mutant strain that were used in the assay. The phagocytic index for each mutant was determined by calculating the number of internalized cells per macrophage and normalizing this to the value for WT KN99 and the number of fungi added. An uptake score was then calculated for each well as fold-change of the normalized phagocytic index compared either to the plate median (for the initial screen of Madhani library plates) or to WT (for the follow-up screens with BMDM and HMDM). For the initial screen of the Madhani deletion collection, we calculated the first quartile (Q1), third quartile (Q3), and interquartile range (IQR) for each plate. We defined hits as those below Q1-c*IQR or above Q3-c*IQR, where c was calculated using a targeted error rate, α, of 0.01. For the second round of screening in BMDM and HMDM, we used an internal WT control and defined hits as those strains whose uptake scores differed from WT in a statistically significant way, using Dunnett's 1-way ANOVA and a p value cutoff of 0.05.

Capsule screen

Strains identified as hits in the uptake assay and wild type control cells were cultured in YPD from frozen stocks in glycerol/YPD in 96 well format (ON, 30 °C, 800 rpm); 10 µL aliquots were then sub-cultured into 190 µL fresh YPD and grown for an additional 24 h. Cells were then sedimented (3000 g, RT) and resuspended in 200 µL DMEM. To achieve the low cell density necessary for optimum capsule induction, the average OD_{600} of the plate was used to calculate the volume of cells to resuspend in 150 µL DMEM to achieve an average OD_{600} of 0.01. Capsule induction was performed in a 96-well glass-bottom plate coated with poly-lysine (Eppendorf) and incubated for 24 h at 37°C with 5% CO_2. KN99α cells grown

overnight in YPD were added to empty wells 10 min before the induction ended to serve as an uninduced control. Following induction, cells were stained and imaged as published previously (32). Briefly, the capsules were stained with anti-capsular monoclonal antibody 302 conjugated to Alexa Fluor 488 (Molecular Probes) and the cell walls stained with Calcofluor White (MP Biomedicals). Imaging was performed on a BioTek Cytation 3 imager collecting 64 images per well in an 8x8 grid pattern. Images were prepared, annotated, and cell wall and capsule diameters for annotated cells calculated as described previously (32). Capsule thickness was defined as the difference between cell wall and capsule diameters. A negative control value (mean capsule thickness of WT cells grown in YPD in the same plate) was subtracted and the result normalized to the value for similarly-corrected positive control samples (WT cells induced in the same plate) to yield a capsule score. Hypocapsular and hypercapsular hits were categorized as those with capsule scores of less than 0.5 or more than 2.0, respectively.

<u>Gene ontology enrichment analysis</u>

Gene ontology (GO) analysis was performed using FungiDB, using both manually curated and computationally predicted GO terms (29, 30, 52). GO categories that were enriched with a P-value cutoff of 0.01 were manually selected for each screen to minimize over-representation of redundant phenotypes.

<u>Strain construction and validation</u>

sac1Δ, *SAC1* complement, and *SAC1*^{D456A} strains were engineered using biolistic transformation with a split-marker strategy (70) in a KN99α background. Design of the catalytically-inactivated construct was based on alignment (36) to the *S. cerevisiae* S288C Sac1 amino acid sequence. mNeonGreen-tagged strains were generated using pBHM2404, a gift from Hiten Madhani (Addgene 173441). PI4P-binding constructs were generated using pRS406-PHO5-GFP-hFAPP1 PH domain, a gift from Tim Levine (Addgene 58723) (44). This sequence was placed between a *TEF1* promoter and terminator and inserted into the Safe Haven 2 (SH2) region of the genome (72). All fluorescent constructs were generated using CRISPR with short homology-directed repair (71). Transformants were confirmed by appropriate antifungal resistance, PCR, and whole genome sequencing.

To test complementation of *sac1*Δ by the corresponding *S. cerevisiae* sequence, the *SAC1* gene was amplified from *S. cerevisiae* strain S288C and inserted into plasmid pMSC042-neo between an *ACT1* promoter and *TRP1* terminator. This construct (containing the promoter, *SAC1* sequence, terminator and antifungal resistance marker) was inserted into the SH2 region of WT and *sac1*Δ cells using CRISPR (71, 72). Total RNA was extracted from cells grown in YPD and DMEM using

TRIzol Reagent (Thermo Fisher) and cDNA was synthesized using the SuperScript III First-Strand Synthesis SuperMix kit (Thermo Fisher). PCR amplification of an S288C Sac1-specific sequence from the cDNA confirmed expression of the S288C *SAC1* gene in both backgrounds (Fig. S1B).

Fungal cell growth and capsule induction

C. neoformans strains were grown from glycerol stocks on YPD plates for 2 days at 30°C. Overnight cultures were inoculated from single colonies into 4 mL of YPD medium and grown overnight at 30°C with shaking (unless otherwise noted). For all capsule induction experiments, overnight cultures were washed in sterile PBS, inoculated into DMEM at 10^6 cells/mL, and grown statically at 37°C with 5% CO_2 for 16-24 hours (as indicated in the text).

Virulence studies

All animal protocols were approved by the Washington University Institutional Animal Care and Use Committee (Protocol #20-0108) and care was taken to minimize animal handling and discomfort. 6-8-week old female C57BL/6 mice (The Jackson Laboratory) were anesthetized by subcutaneous injection of 1.20 mg ketamine and 0.24 mg xylazine in 110 µL sterile PBS. For 14-day infections, mice were intranasally infected with 5×10^4 cryptococcal cells. For time course infection, mice were infected with 1.25×10^4 cryptococcal cells and sacrificed at 6-, 12-, and 18-days post infection. For both infections, the lungs and brains were harvested, homogenized, and plated on YPD agar for calculation of fungal organ burden.

Stress plate dot spotting

Overnight cultures were pelleted and washed twice in PBS, then diluted to 10^7 cells/mL. 4 µL of this stock and four serial 1:10 dilutions were spotted onto YPD agar and incubated at both 30 and 37°C for 48 hours. To impose plasma membrane stress, the YPD was supplemented with 0.01% sodium dodecyl sulfate or 1 µg/ml amphotericin B; to impose cell wall stress, the YPD was supplemented with 0.05% Congo red.

Acid phosphatase secretion assay

Single colonies were inoculated into 5 mL YPD, grown shaking at 30°C for 6 h, sub-cultured into 25 mL MM-KH_2PO_4 (0.5% KH_2PO_4, 15 mM glucose, 10 mM $MgSO_4{\cdot}7H_2O$, 13 mM glycine, 3.0 µM thiamine), and grown overnight at 30°C. Cells were then pelleted, washed twice with MM-KCl (0.5% KCl, 15 mM glucose, 10 mM $MgSO_4{\cdot}7H_2O$, 13 mM glycine, 3.0 µM thiamine), and resuspended in 50 mL MM-KCl at 10^7 cells/mL. Cultures were grown at 30°C with shaking, and 2 mL aliquots were collected at each timepoint. For each, pelleted cells were resuspended in 400 µL substrate buffer (2.5 mM para-

nitrophenylphosphate in 50 mM acetate buffer, pH 4.0, Sigma 71768-5G), incubated at 37°C for 5 minutes, and the reaction stopped with 800 µL saturated Na_2CO_3. Acid phosphatase secretion was quantified using absorbance at 420 nm, corrected by subtraction of a cell-free blank value, and normalized to cell density using OD_{600}.

<u>GXM ELISA</u>

Cultures were induced in DMEM for the indicated times, at which point cells were removed by centrifugation (3000 x g, 5 min, RT), the supernatant fraction filtered through a 0.2µm filter, and the filtrate diluted 1:500 in PBS. Flat-bottom Immulon 1B microtiter plates (Thermo Scientific) were coated overnight with a 1 µg/ml mix of anti-GXM antibodies 339 and F12D2γ in PBS. This and the following incubations were performed at room temperature. The plates were washed twice with 200 µL PBS with 0.5% TWEEN 20 (PBST), blocked for 90 min with 200 µL 1% BSA in PBST. The blocking buffer was replaced with 100 µL PBST and 100 µL of filtered sample was added and incubated for 90 min. The plate was then washed three times as above and the bound GXM was probed with HRP-labeled mAb 339/F12D2γ, incubated for 1 minute with KPL TMB Microwell Peroxidase Substrate (SeraCare), developed, and quantified using absorbance at 450 nm.

<u>Imaging</u>

For fluorescence and confocal microscopy, cells were grown as indicated above and washed twice in PBS. For capsule visualization, cells were resuspended in India ink in PBS (1:2, v:v) and imaged on a ZEISS Axio Imager M2 fluorescence microscope. Capsule thickness was measured manually in ImageJ, using a total of at least 96 cells from at least six randomly-chosen fields of view. To stain neutral lipids, a 0.01% Nile Red stock in acetone was diluted to a working concentration of 0.0005% in PBS. Cells were incubated in 1 mL working Nile Red solution for 15 min in the dark, rotating. Cells were washed twice in PBS, resuspended in PBS, and imaged as above. For quantification of morphology, a minimum of 60 cells from randomly-chosen slide coordinates were examined. For filipin staining, cells were incubated in a freshly prepared solution of 5 µg/mL filipin (Millipore Sigma, F9765-25MG) in DMSO for 15 minutes at room temperature, rotating, then washed twice with PBS before imaging as above. To visualize FAPP1-mNeonGreen and Cxt1- mNeonGreen, cells were imaged using a Zeiss LSM880 confocal microscope. For quantification of morphology, a minimum of 94 cells from randomly-chosen slide coordinates were examined. For electron microscopy, cells were grown overnight in YPD or induced for 24 hours in DMEM before processing as in (50), except that the first incubation in 0.1 M sodium cacodylate buffer was performed for 2 h at RT.

<u>Lipid supplementation</u>

For lipid supplementation, a fatty acid stock solution (10 mM in ethanol) was diluted 1:10 into 4 mg/ml BSA in DMEM and then sonicated (Bransonic 2 Ultrasonic Bath Sonicator) before addition to cultures as indicated. After 24-hour of incubation (37°C, 5% CO_2), cells were serially diluted 1:10 and plated onto YPD agar, with the top spot representing the undiluted culture.

GXM purification and analysis

GXM was isolated using selective precipitation of culture supernatants with hexadecyltrimethylammonium bromide (CTAB) as described previously (51). Isolated material was hydrolyzed in trifluoroacetic acid for 3 hours at 100°C and compositional analysis performed using high-performance anion-exchange chromatography with pulsed amperometric detection on a Dionex ICS-6000 instrument (Thermo Scientific). Glycosyl linkage analysis was performed by the Complex Carbohydrate Research Center at the University of Georgia using combined gas chromatography/mass spectrometry of partially methylated alditol acetate derivatives following carboxyl reduction to detect uronic acids (modified from (75)).

Supplemental Table Legends

Supplemental Table S1. Uptake screen results. (S1A) All results from the initial BMDM uptake screen. (S1B) Hits from the initial BMDM uptake screen. (S1C) All results from the second BMDM uptake screen. (S1D) Hits identified from the second BMDM uptake screen. (S1E) All results from the HMDM uptake screen. (S1F) Hits from the HMDM uptake screen.

Supplemental Table S2. Capsule screen results. (S2A) All results from the capsule screen. (S2B) Hits identified from the capsule screen. (S2C) Overlap hits identified in all three screens: HMDM, BMDM, and capsule.

Supplemental Table S3. Linkage analysis of WT, *sac1*Δ, and *SAC1* strains. Values are the percent area of relevant residue peaks. Numbers in the header row refer to independently isolated samples.

Supplemental Figure Legends

Supplemental Figure S1. Overexpression of the *S. cerevisiae* Sac1 protein in *sac1*Δ does not restore *C. neoformans* capsule synthesis. (A) Negative stain of the indicated strains grown for 24 hours in DMEM at 37°C with 5% CO_2, with the capsule thickness in µm (mean ± SD). Scale bar, 10 µm. (B) Amplification of a unique 651 bp region of the S288C Sac1 coding sequence from cDNA generated from fungal cells of the indicated strain grown in YPD shows that the *S. cerevisiae SAC1* gene is expressed in both *C. neoformans* strains.

Supplemental Figure S2. Construction and validation of the $SAC1D^{456A}$ strain. (A) Strain construction strategy for *SAC1* complement and SAC^{D456A} inactivated complement strains. (B) Normalized expression of *SAC1* in WT and $SAC1^{D456A}$ cells grown for 24 hours in DMEM at 37°C with 5% CO_2. (C) Nucleotide sequence of $SAC1^{D456}$. Red, nucleotide differences from WT *SAC1*.

Supplemental Figure S3. Cells lacking functional Sac1 have reduced virulence. (A) Lung and (B) brain burden of C57BL/6 mice sacrificed at the indicated days post infection (dpi) with 1.25×10^4 fungal cells of *ckf44_06080*Δ from the Madhani *C. neoformans* deletion collection. Upper dotted line, inoculum. Lower dotted line, limit of detection. Each symbol represents one mouse.

Supplemental Figure S4. PI4P content of WT and *sac1*Δ strains. Difference in median fluorescence intensity between FAPP1-mNG expressing and control cells (ΔMFI) in WT and mutant backgrounds, grown in the indicated medium. ns, not significant by unpaired t test.

Supplemental Figure S5. Dual staining with filipin and Nile Red. All cells were grown in DMEM for 24 hours at 37°C with 5% CO_2. DIC, Differential interference contrast. Scale bar, 10 µm.

Supplemental Figure S6. Supplementation with exogenous fatty acids partially restores growth of *sac1*Δ cells. Serial dilutions of (left) WT or (right) *sac1*Δ cells grown for 24 hours in DMEM alone, DMEM supplemented with BSA, or DMEM with 100 µM of the indicated fatty acid sodium salt conjugated to 0.4 mg/mL BSA.

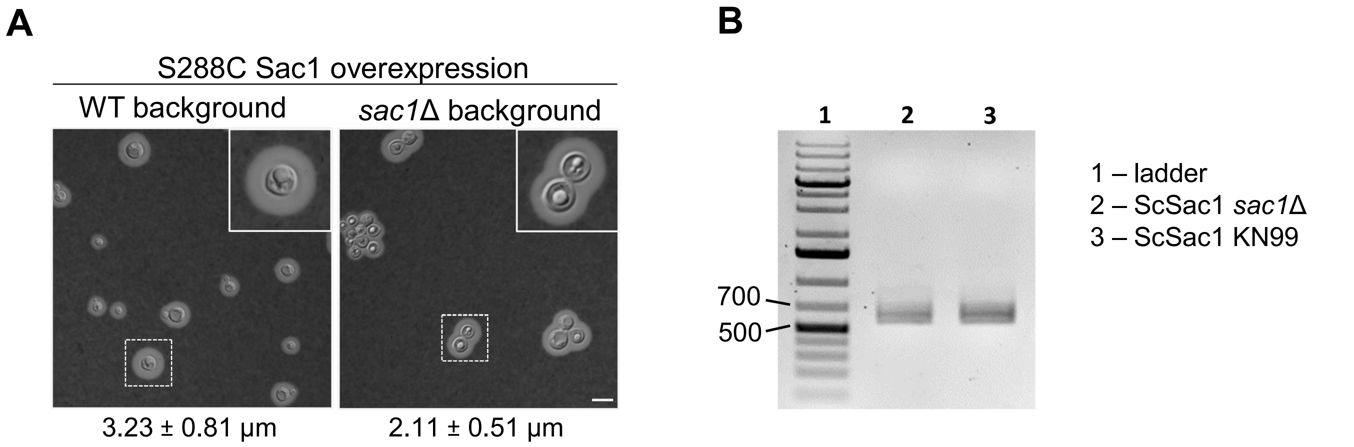

Supplemental Figure S1. Overexpression of the *S. cerevisiae* Sac1 protein in *sac1*Δ does not restore *C. neoformans* capsule synthesis. (A) Negative stain of the indicated strains grown for 24 hours in DMEM at 37°C with 5% CO_2, with the capsule thickness in μm (mean ± SD). Scale bar, 10 μm. **(B)** Amplification of a unique 651 bp region of the S288C Sac1 coding sequence from cDNA generated from fungal cells of the indicated strain grown in YPD shows that the *S. cerevisiae SAC1* gene is expressed in both *C. neoformans* strains.

A

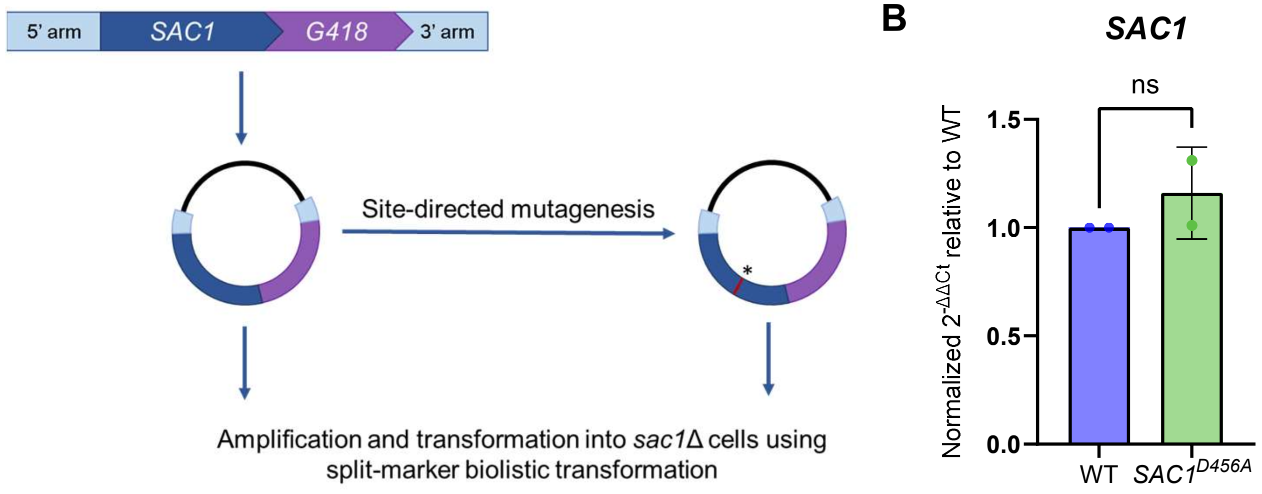

C

ATGGCGTTTAATGCTCTACCTGTATGTGACCTTCGCGAAGTGAACTGGCTGCCGCTAACAAGACTTAGCTTCACGAAACC
TTAAACCTCTACGTCTCTCCTACGGCTTACATTTTCGAGCCTGCTTCCTCCAGTGCTGGCCATGTGGGAATCGATGGAAC
TATATTCGTTGATGAAAAGAATGTAAGGGAGAGTATGGTTGTGGACCGTCAGACTGGCCAGATTAGCTTGAGTAGTGAGT
GACAACCCTTGCAATGCGTGGAGCAGAATATCACTGATGCGACAGAACATAACAGCGGCTTCTCATATACCATTTGGAAA
GGAAAAGGTTATTACTTGCTACGGTATTATTGGCATTCTCAGCCTTGCTACTAGTAAGTTTTGTTGCTCCTCAGACACAA
TTGCCCCCTGCTGATTTTGTGCTTTCTAATAGCCGATTTTCTTCTTATTGTCACTTCCCGCTCTCCTTCATGTCGCCTTC
TTTCTCACCCTATCTATCTTGCGAATGATTATCGACTTCTTCCTATTTCCCCTCTGTCTACTTCCTCCGCCATTCTTGAA
CATCCTGTGGAGAAGGAACTCATCTCTCTTGTTGAGCAGGGTCTCAAATCCAGTAAACTCTGGTTCTCGTATGGTTGGGA
CCTTACCAATTCTTTGCAGAGGCAACAAGAAATCGATTTGAAGCTCTCACAGTCCGGAGAACAATGGCCCGCCTGGAGAC
GGGCAGATGAACGTTTCTTTTGGAACAGGTTTCTCATGGACAAGATGATTGACGTGACCGAGAGCGGTGAGGCAGATCTG
AGCCGATTTATCCTTCCTATAATGTACGGCTCTATTGAGCTTCGGTCATCGACTCTTAACTCTCGGGATCTCCTTTTCCT
ACTCATCTCTCGGCGATCTCGTTACCGAGCTGGTACCAGATACTTCACGCGAGGCATCAACCCCTCTGGCCACGTCGCCA
ATTTCAACGAGACTGAGCAAATTGTTATGTATGACCCTATCCCGGAGAACGGAGAAGCCTACGGAAGGGGAAGGGTAGAC
GGTAGGGAAAGGCTGAGCTTTGTGCAGACAAGAGGGAGTGTTCCGCTATTCTGGGCCGAGGTCAACAACTTGAGGTACAA
GCCCGATTTGCAAATTATGGATTACACTGAGACGGTGAGTGAATTGTATTGAAAGGCATGACATGTCCTGACAATGAATT
AGCCTCAAGCTCTCAAAGCACATCTCAACAGTATGATTAAGACCTATGGCCATACATATCTTGTGAATCTTGTAAATCAA
AAGGGACACGAACAACCTGTCAAGGAAGCTTTTGAGCGCTACATGTCCCTTGTCGCCTCATCCGACCCGTCCATTCAGGA
GAAGGCTCACTACCTCTACTTTGACTTTCACCACGAATGCAAAGGTCTAAGGTTTGACCGAATTTCCCTTTTGGTCGAGA
AGCTTGCGACAGCACTTGAGGATATGAGCTGGTACCATTCTGTCAACCCCGACTCGTCAACCTATGCTCTCTTGCAACCA
AACTCTGCAGACACCCAGGTACTTACTAAGCAAACGGGCGTCGTCAGAACCAACTGTATGGCGTGTCTAGACAGGACCAA
TGTCGCACAAGCGGCTCTGGCTAGATGGGTTTTGAACCGGCAATTAAGGAAGGTCGGGATCCTGAGCGTGAAGGAAGGTG
TGGAGGATCACGAAGAGTTTATGATTATGTTCCGGAATGGTACGTGCTTTTCATTTTGACTAGAAATTAGACTAACTTGA
GAAAATAGTGTGGGCCGATCATGGTGACACCGTCTCCCGAGCTTATGCAGGAACTGGTGCTCTCAAATCCGACTTTACTC
GTACCGGTAAACGATCCAAGGAGGGTCTCTTGGAAGATGGTTACAAAAGCGTGATGAGATACTTTAGGAATAACTTCTTC
GATGGCGATCGTCAGGTTAGTCCCTACTGTTGTGAGCTGAGAGAGACATCTGACCGGAGAACAGGATGGTTTTGATATTC
TAACTGGAGCATGGGTAGCCCAAAGGGGTGGCATTCCTCCATTGACTGATACAAGACCTCTTATCATGCGCTCGGTAAGT
CCATGGTTTGTCCGACTAATGTTGACCCGAGAAGATGCCGTACATACTGGCGTTTGCTCTCACAATGATCACCGCAGCTC
TCACATTGCCACGAACCTCGGAGATGTCCATATATTCCTTCCTCGTCCTTTGGTTCTTCCTTGCATTCTTCTCAGGAAGT
TATATTTGGGGCAATGTAAGTTATTTTCTTCTTTCATATATTGACTTTGGCCGTTGACCGCCATTTGTCACTCTTAGGGT
ACATCATACGTCTCCTGGCCGCGTTTGAACCCTCCTCTAGAAATTCTCTCTTACTCTGGACCGGGTCACCGCTCTCCGGT
CCGCGGTCGAGGGATGTCGTTCGCAGCCATTGTGCCCCTGTTCGATCGTCGACGCCAAAATGGTCCTGGAGCTGGCGTAC
TTGGTGCCACCGGCGGCTTCGGAGGAAGGAACGATGGAAGACCTGCAATGTATAAGATGGAGGAGTCGGAGTTGGGGAGG
AGGAAGGGTGCGTTGATTGATTAA

Supplemental Figure S2. Construction and validation of the *SAC1D*^{456A} strain. **(A)** Strain construction strategy for *SAC1* complement and *SAC*^{D456A} inactivated complement strains. **(B)** Normalized expression of *SAC1* in WT and *SAC1*^{D456A} cells grown for 24 hours in DMEM at 37°C with 5% CO_2. **(C)** Nucleotide sequence of *SAC1*^{D456A}*.* Red, nucleotide differences from WT *SAC1*.

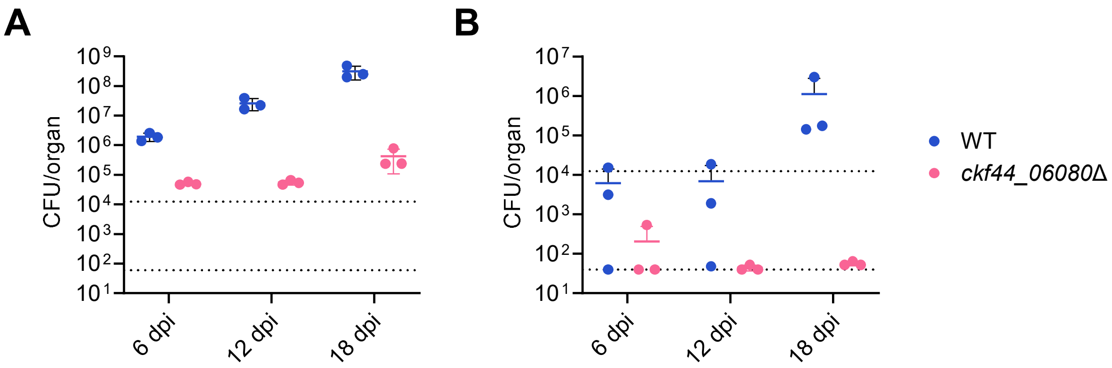

Supplemental Figure S3. Cells lacking functional Sac1 have reduced virulence. (A) Lung and **(B)** brain burden of C57BL/6 mice sacrificed at the indicated days post infection (dpi) with 1.25×10^4 fungal cells of *ckf44_06080Δ* from the Madhani *C. neoformans* deletion collection. Upper dotted line, inoculum. Lower dotted line, limit of detection. Each symbol represents one mouse.

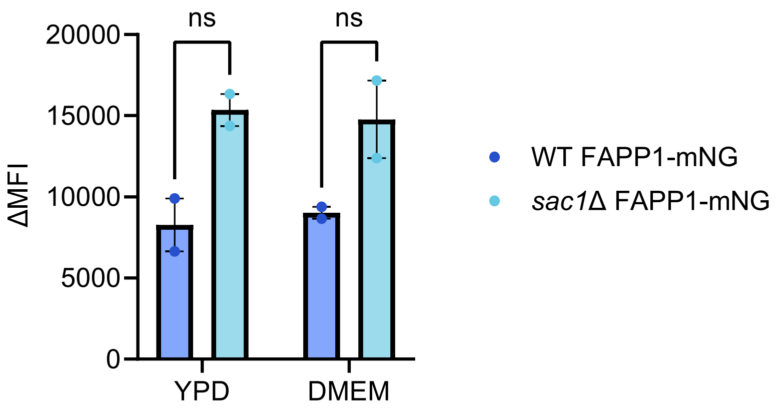

Supplemental Figure S4. PI4P content of WT and *sac1*Δ strains. Difference in median fluorescence intensity between FAPP1-mNG expressing and control cells (ΔMFI) in WT and mutant backgrounds, grown in the indicated medium. ns, not significant by unpaired t test.

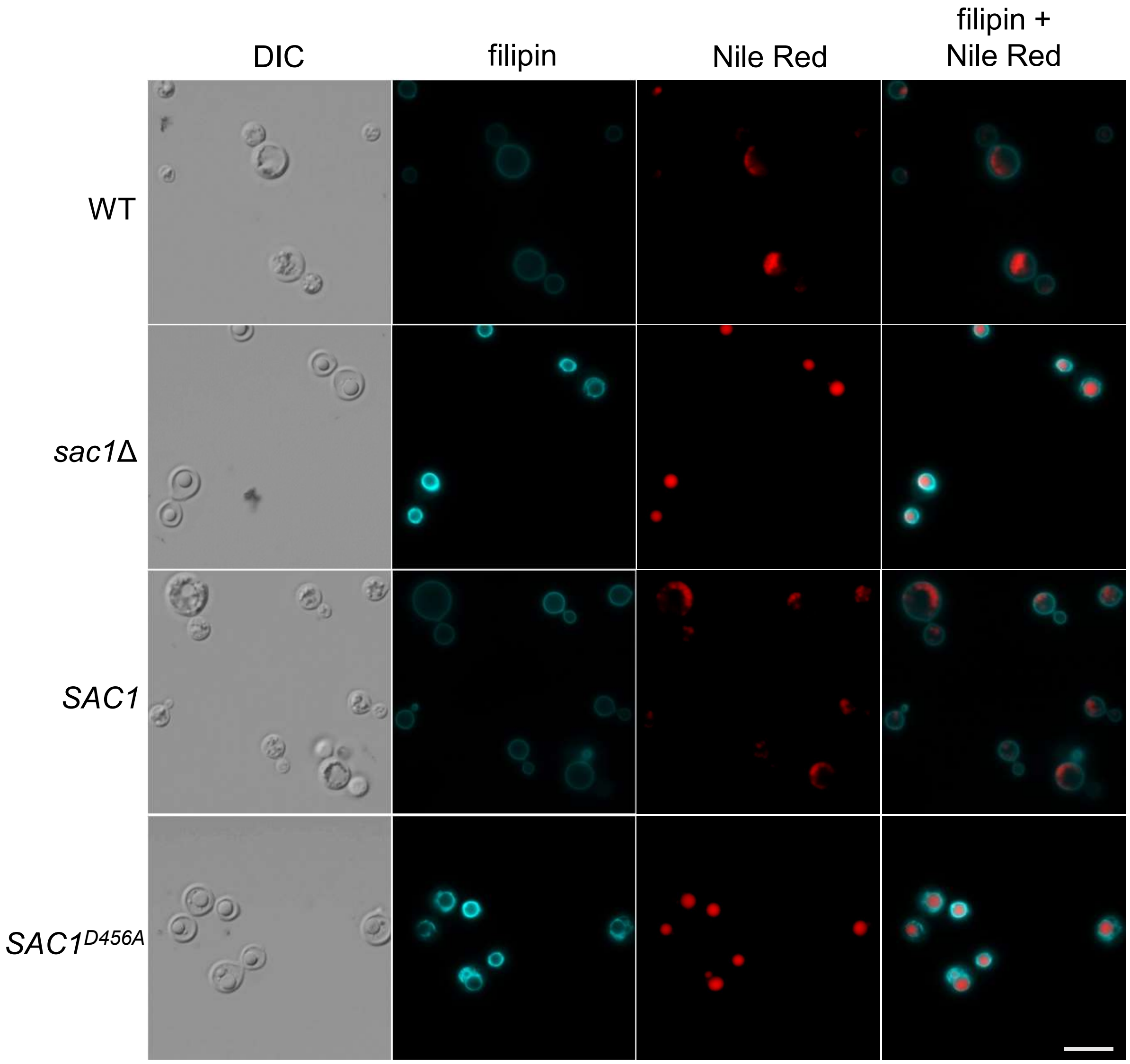

Supplemental Figure S5. Dual staining with filipin and Nile Red. All cells were grown in DMEM for 24 hours at 37°C with 5% CO_2. DIC, Differential interference contrast. Scale bar, 10 μm.

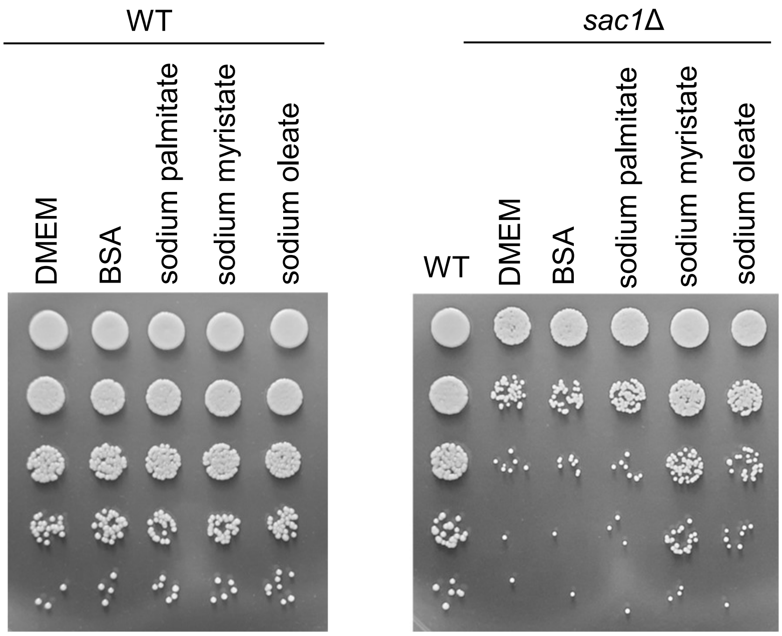

Supplemental Figure S6. Supplementation with exogenous fatty acids partially restores growth of *sac1*Δ cells. Serial dilutions of (left) WT or (right) *sac1*Δ cells grown for 24 hours in DMEM alone, DMEM supplemented with BSA, or DMEM with 100 μM of the indicated fatty acid sodium salt conjugated to 0.4 mg/mL BSA.

Residue	WT #1	*sac1*Δ #1	WT #2	*sac1*Δ #2	*SAC1* #2
Terminal Xylopyranosyl residue (t-Xyl)	12.0	19.7	10.4	16.2	11.1
Terminal Mannopyranosyl residue (t-Man)	1.3	0.3	0.3	0.3	0.4
Terminal Glucopyranosyl uronic acid residue (t-GlcA)	17.9	15.4	19.1	16.2	18.2
3-linked Mannopyranosyl residue (3-Man)	29.9	23.3	27.8	21.1	30.6
2,3-linked Mannopyranosyl residue (2,3-Man)	36.0	38.5	37.5	42.8	34.9
2,3,4-linked Mannopyranosyl residue (2,3,4-Man)	2.9	2.8	4.9	3.5	4.8

Supplemental Table S3. Linkage analysis of independently isolated WT, sac1Δ, and *SAC1* strains. Values are percent area of relevant residue peaks. Numbers in header row refer to independently isolated samples.
