# Supplementary material for: Sac1 links phosphoinositide turnover to cryptococcal virulence
Source: mBio. 2024 Jul 2;15(8):e01496-24. doi: 10.1128/mbio.01496-24 (PMC11323556; doi:10.1128/mbio.01496-24)
Supplement: Table S3 — Linkage analysis. [file mbio.01496-24-s0004.docx]

| Residue | WT #1 | *sac1*Δ #1 | WT #2 | *sac1*Δ #2 | *SAC1* #2 |
| --- | --- | --- | --- | --- | --- |
| Terminal Xylopyranosyl residue (t-Xyl) | 12.0 | 19.7 | 10.4 | 16.2 | 11.1 |
| Terminal Mannopyranosyl residue (t-Man) | 1.3 | 0.3 | 0.3 | 0.3 | 0.4 |
| Terminal Glucopyranosyl uronic acid residue (t-GlcA) | 17.9 | 15.4 | 19.1 | 16.2 | 18.2 |
| 3-linked Mannopyranosyl residue (3-Man) | 29.9 | 23.3 | 27.8 | 21.1 | 30.6 |
| 2,3-linked Mannopyranosyl residue (2,3-Man) | 36.0 | 38.5 | 37.5 | 42.8 | 34.9 |
| 2,3,4-linked Mannopyranosyl residue (2,3,4-Man) | 2.9 | 2.8 | 4.9 | 3.5 | 4.8 |
